# Comparison of dural grafts and methods of graft fixation in Chiari malformation type I decompression surgery

**DOI:** 10.1038/s41598-021-94179-4

**Published:** 2021-07-20

**Authors:** Artur Balasa, Przemysław Kunert, Tomasz Dziedzic, Mateusz Bielecki, Sławomir Kujawski, Andrzej Marchel

**Affiliations:** 1 Department of Neurosurgery, Medical University of Warsaw, ul. Banacha 1a, 02-097 Warsaw, Poland; 2 Division of Ergonomics and Exercise Physiology, Department of Hygiene, Epidemiology, Ergonomics and Postgraduate Training, Collegium Medicum in Bydgoszcz, Nicolaus Copernicus University in Torun, M. Sklodowskiej-Curie 9, 85-094 Bydgoszcz, Poland

**Keywords:** Diseases, Outcomes research, Neurological disorders

## Abstract

Suboccipital decompression with duraplasty is a widely accepted method for treating patients with Chiari malformation type I. However, important details of the duraplasty technique are still controversial. This retrospective study analyzes clinical and radiological outcomes after surgery depending upon the type of graft and methods of graft fixation. Seventy consecutive decompressions with duraplasty were analyzed. Two types of grafts, nonautologous (Non-AutoG; 60.0%) and autologous (AutoG; 40.0%), and two methods of graft fixation, suturing (S; 67.1%) and gluing (G; 32.9%), were used in four different combinations: (Non-AutoG+S: 31.4%; Non-AutoG+G: 28.6%; AutoG+S: 35.7%; AutoG+G: 4.3%) according to surgeon preference. The mean follow-up was 63.4 months. According to gestalt and Chicago Chiari Outcome Scales, satisfactory results were obtained in 72.9% and 78.6% of cases, respectively, in the long term. The outcomes were not related to the kind of graft (p = 0.44), fixation method (p = 0.89) or duraplasty pattern (p = 0.32). Decreased syringomyelia was observed in 88.9% of cases, and no associations with the kind of graft (p = 0.84), fixation method (p = 1) or duraplasty pattern were found (p = 0.96). Pseudomeningocele occurred 5 times more often in the Non-AutoG group than in the AutoG group (52.4% vs. 10.7%; p < 0.05), whereas their formations were not related to the fixation method (p = 0.34). Three cases (12.0%) required reoperation with reduraplasty. Autologous and nonautologous dural grafts can be sutured or glued with similar clinical results; however, the use of nonautologous grafts is linked with a much higher risk of pseudomeningocele formation.

## Introduction

Chiari malformation (CM) was described for the first time by pathologist Hans Chiari in 1891 and refers to congenital caudal displacement of the hindbrain elements through the foramen magnum. The prevalence of CM is estimated as ranging from 0.24 to 3.6% of the population with a slight female predominance^1–3^. The treatment of choice in symptomatic cases is posterior fossa decompression, described for the first time in 1938 by Penfield^4^. This procedure has changed and improved over the years. Suboccipital craniectomy with C1 or even C2 laminectomy and duraplasty is currently a widely accepted and recommended method for treating patients with CM^5^.

Different kinds of material are used for duraplasty, including autologous tissues such as epicranial aponeurosis or muscle fascia and nonautologous (synthetic) materials such as bovine collagen matrix^6–8^. Details of the surgery, especially concerning the duraplasty techniques, are still the subject of debate^9–12^. However, to our knowledge, there has been no study evaluating the kind of graft used for duraplasty along with the method of its fixation. The aim of our study was to compare the long-term clinical and radiological outcomes of surgery for CM-I, together with a complication analysis, depending upon the duraplasty materials and methods of graft fixation.

## Materials and methods

Ninety patients with symptomatic Chiari malformation type I (CM-I) underwent posterior fossa decompression (PFD) with duraplasty from January 2003 to December 2018. Twenty patients were lost to radiological follow-up and were excluded from the study. Finally, 70 patients were included. The whole group involved 54 women and 16 men, ranging in age from 18 to 66 years (average 41.9 years old). The average duration of symptoms was 67.3 months (range: 3 months–50 years). The main reasons for diagnosis were suboccipital or general headache (68.6%) (Table 1). The mean follow-up was time 63.4 months (range 3–187 months).

**Table 1:** Preoperative signs and symptoms in 70 patients with symptomatic Chiari malformation type I.

Signs and symptoms	Number of patients (%)
Suboccipital or general headache	48 (68.6%)
Neck and back pain	45 (64.3%)
Shoulder and arm pain	28 (40.0%)
Decreased temperature sensitivity	32 (45.7%)
Decreased touch sense	22 (31.4%)
Decreased proprioceptive sense	3 (4.3%)
Decreased pain sensitivity	25 (35.7%)
Somatosensory disturbances (paresthesia, hyperesthesia)	37 (52.9%)
Paresis or motor weakness	30 (42.9%)
Cerebellar signs (nystagmus, ataxia, dizziness, dysarthria, imbalance)	28 (40.0%)
Dysphagia	15 (21.4%)
Visual disturbance	3 (4.3%)
Sleep apnea	0 (0.0%)
One-sided signs or symptoms	23 (32.9%)
Both-sided signs or symptoms	34 (48.6%)
Non-specified	13 (18.6%)

All patients underwent suboccipital craniectomy with C1 posterior arch removal. Partial or whole C2 laminectomy was additionally performed in 24 (34.3%) and 6 (8.6%) patients, respectively. The range of cervical bony decompression depended upon the level of tonsil descent.

Two types of grafts were used: nonautologous (Non-AutoG) in 42 patients (60.0%) and autologous (AutoG) in 28 cases (40.0%). Non-AutoG included Duragen Integra (22), Duragen Plus Integra (12), Durepair Medtronic (8) and AutoG included fascia lata (4) or pericranium (24).

**Figure 1:**
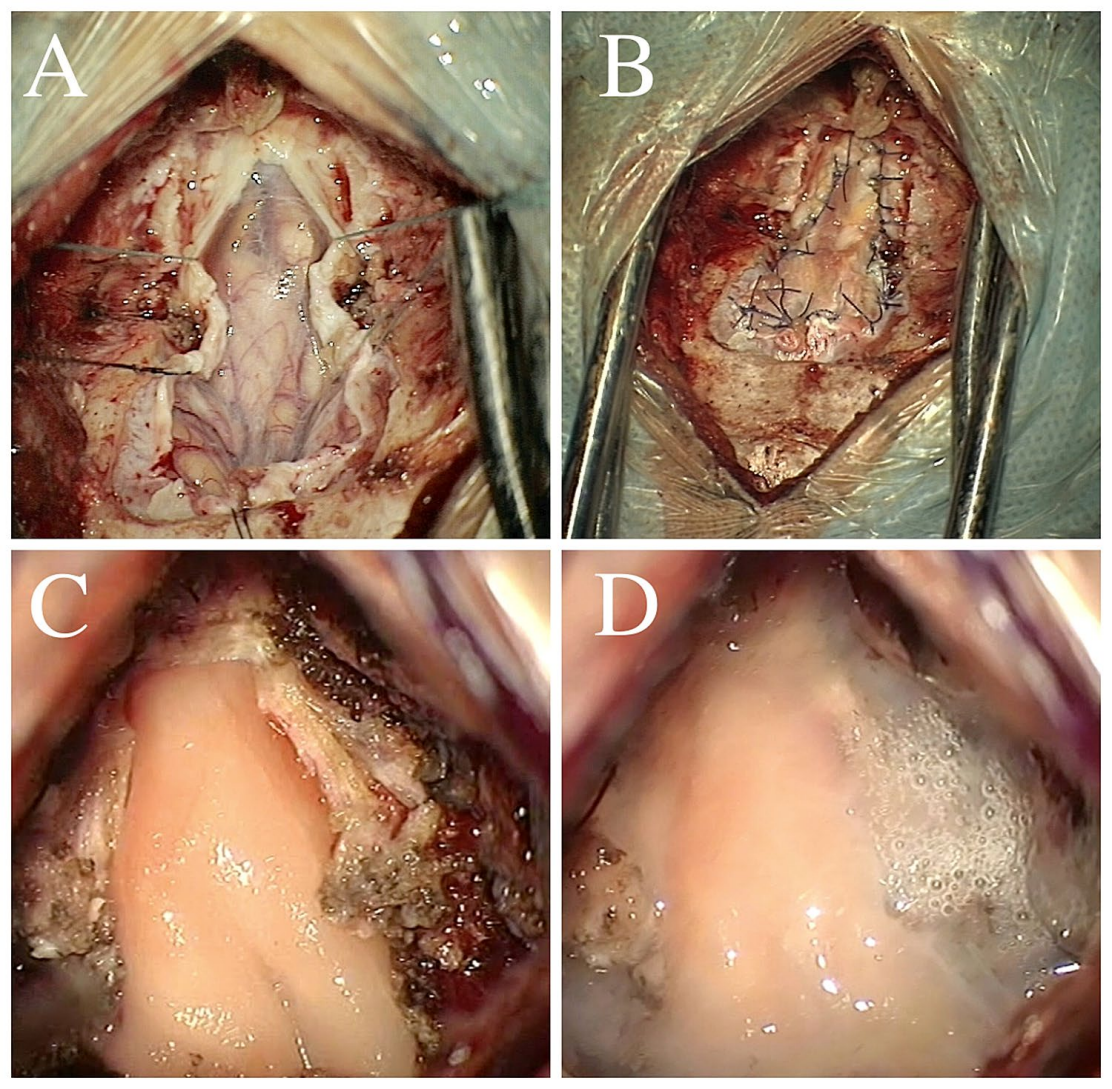
Intraoperative photographs showing two of the four duroplasty patterns described in article. (**A**) Bony decompression, Y-shaped incision of the dura with arachnoid membrane intact. (**B**) Autologous graft (pericranium) sutured in a watertight fashion (AutoG+S). (**C**,**D**) Nonautologous graft (Duragen) fixed with fibrin glue only (Non-AutoG+G).

The grafts were fixed in two ways: with sutures (S) in 47 (67.1%) cases or with fibrin glue (G) only in 23 (32.9%) cases. Therefore, 4 different patterns of duraplasty were distinguished: (1) nonautologous graft fixed with sutures (Non-AutoG+S; 31.4%), (2) nonautologous graft fixed with glue (Non-AutoG+G; 28.6%), (3) autologous graft fixed with sutures (AutoG+S; 35.7%), and 4. autologous graft fixed with glue (AutoG+G; 4.3%). The extent of decompression depended upon the level of tonsil descent, but the duraplasty technique was related to surgeon preference. Patients were operated on using a consistent surgical technique, i.e., without opening the arachnoid and 4th ventricle or tonsil resection (see Fig. 1).

Patients were divided into groups depending on surgical technique and the presence of pseudomeningocele on follow-up MRI. Long-term clinical outcomes were evaluated with gestalt assessment (improvement, unchanged or deterioration) and the Chicago Chiari Outcome Score (CCOS, range 4–16 score)^13,14^. Based on gestalt, we divided patients into satisfactory (improvement and unchanged) and unsatisfactory (deterioration) groups. Similarly, using the CCOS, the patients were assigned to the satisfactory (≥ 12 score) or unsatisfactory (< 12 score) group. The size of the syrinx cavity was compared to that on preoperative MRI and evaluated as decreased, stable or increased. All complications requiring separate management were recorded and analyzed as potentially dependent upon the duraplasty technique. In follow-up MRI, the presence and average maximal thickness of pseudomeningoceles was evaluated within each duraplasty group.

According to the statement of Bioethics Committee of Medical University of Warsaw this study did not require IRB/ethics committee approval and patient consent. All methods were carried out in accordance with relevant guidelines and regulations.

### Statistical analysis

The aligned rank transform tool (ARTool package) for nonparametric factorial ANOVAs in the R environment was used with a group factor and time factor. Differences between more than two groups were analyzed using Kruskal–Wallis one-way analysis of variance with the Dwass-Steel-Critchlow-Fligner test and *Benjamini and Hochberg* adjustment of the p value for post hoc tests. Qualitative variables were analyzed using the chi-squared test with appropriate correction applied^15,16^.

## Results

The mean preoperative symptom duration in the Non-AutoG, AutoG, S and G groups was 67.5, 67.1, 73.4 and 54.7 months, respectively (Non-AutoG vs. AutoG: p = 0.23; S vs. G: p = 0.11). The mean symptom durations in the Non-AutoG+S, Non-AutoG+G, AutoG+S and AutoG+G groups were as follows: 79.6, 53.3, 68.0 and 64.0 months (p = 0.11).

Based on gestalt and CCOS in the whole cohort, satisfactory results were obtained in 72.9% and 78.6% of patients, respectively (mean CCOS 12.34; SD ± 2.41). Satisfactory outcomes on the gestalt scale were observed in 76.2% of the non-AutoG group and in 67.9% of the AutoG group (p = 0.44). The rates of satisfactory CCOS (≥ 12) were identical in the Non-AutoG and AutoG groups (78.6%, p = 1). We observed long-term occipital pain exacerbation in 25.0% of the AutoG group when the autograft was harvested from the same incision and in 9.5% in the Non-AutoG group (p = 0.09).

Considering only the method of graft fixation, satisfactory results based on gestalt and CCOS were observed in 72.3% and 80.9% of the S group and 73.9% and 78.3% of the G group (p = 0.89, p = 0.79), respectively (Table 2).

**Table 2:** Long-term follow-up in Chicago Chiari Outcome Scores (CCOSs) depend on kind of grafts and methods of graft fixation.

CCOS (total score)	16 (excellent outcome)	15-12 (functional outcome)	11-8 (impaired outcome)	7-4 (incapacitated outcome)
Non-AutoG^a^	0 (0.0%)	33 (78.5%)	7 (16.7%)	2 (4.8%)
AutoG^b^	2 (7.1%)	20 (71.4%)	5 (17.9%)	1 (3.6%)
S^c^	2 (4.3%)	34 (72.3%)	9 (19.1%)	2 (4.3%)
G^d^	0 (0.0%)	18 (78.3%)	4 (17.4%)	1 (4.3%)

^1^Non-autologous graft, ^2^Autologous graft, ^3^Graft fixed with sutures, ^4^Graft fixed with glue.

In the Non-AutoG+G group, 80.0% of patients improved or remained unchanged, and 80.0% achieved a satisfactory CCOS. In the AutoG+S group, satisfactory results on the gestalt and CCOS scales were observed in 76.0% and 80.0%, respectively. In the Non-AutoG+S group, satisfactory gestalt and CCOS results were observed in 72.7% and 77.3%, respectively. In the AutoG+G group, 2 out of 3 patients deteriorated on the gestalt scale and had unsatisfactory outcomes in terms of the CCOS.

Fifty-four (77.1%) patients presented with syringomyelia. After surgery, the syrinx length decreased in 88.2% of the Non-AutoG group and in 90.0% of the AutoG group. Regarding the method of graft fixation, the syringomyelia decreased in 88.9% of the S group and 88.9% of the G group (Table 3). The cross-sectional area of the syrinx decreased to a similar extent regardless of the type of graft (p = 0.82) or the method of its fixation (p = 0.78). The mean length of syringomyelia decreased in a similar range (< 2 segments) depending on graft type (p = 0.69) and method of graft fixation (p = 0.44). The syrinx increased in 2 (3.7%) cases on follow-up MRI; both were in the Non-AutoG+S group (9.1%) (Table 4).

**Table 3:** Follow-up summary of clinical and radiological outcomes stratified by the type of grafts used, graft fixation methods and duraplasty materials during decompression surgery in patients with Chiari I malformation.

Kind of grafts, methods of graft fixation and duraplasty pattern	Number of patients	Long-term results						Syringo-myelia present (pre-op) 54/70 (77.1%)	Syringomyelia on follow-up MRI Number of patients (%)			Complication rate			Pseudomeningocele				
		Improvement or unchanged	Deterioration	*p*	CCOS ≥ 12	CCOS < 12	*p*		Decreased 48/54 (88.9%)	Stable 4/54 (74%) or increased 2/54 (3.7%)	*p*	[−]	[+]	*p*	[+]	[−]	*p*	Average maximal thickness^a^ [SD]	*p*
Non-AutoG^1^	42 (60.0%)	32 (76.2%)	10 (23.8%)	0.44	33 (78.6%)	9 (21.4%)	1	34 (80.9%)	30 (88.2%)	4 (11.8%)	0.84	38 (90.5%)	4 (9.5%)	0.80	22 (52.4%)	20 (47.6%)	< 0.05	4.62 (± 5.7)	< 0.05
AutoG^2^	28 (40.0%)	19 (67.9%)	9 (32.1%)		22 (78.6%)	6 (21.4%)		20 (71.4%)	18 (90.0%)	2 (10.0%)		25 89.3%)	3 (10.7%)		3 (10.7%)	25 (89.3%)		0.61 (± 2.1)	
S^3^	47 (67.1%)	34 (72.3%)	13 (27.7%)	0.89	38 (80.9%)	9 (19.1%)	0.79	36 (76.6%)	32 (88.9%)	4 (11.1%)	1	43 (91.5%)	4 (8.5%)	0.87	15 (31.9%)	32 (68.1%)	0.34	2.40 (± 4.4)	0.24
G^4^	23 (32.9%)	17 (73.9%)	6 (26.1%)		18 (78.3%)	5 (21.7%)		18 (78.3%)	16 (88.9%)	2 (11.1%)		20 (87.0%)	3 (13.0%)		10 (43.5%)	13 (56.5%)		4.26 (± 5.9)	
Non-AutoG+S^5^	22 (31.4%)	16 (72.7%)	6 (27.3%)	0.39	17 (77.3%)	5 (22.7%)	0.32	18 (81.8%)	16 (88.9%)	2 (11.1%)	0.96	22 (100.0%)	0 (0.0%)	0.16	12 (54.5%)	10 (45.5%)	< 0.05	4.36 (± 5.4)	< 0.05
Non-AutoG+G^6^	20 (28.6%)	16 (80.0%)	4 (20.0%)		16 (80.0%)	4 (20.0%)		16 (80.0%)	14 (87.5%)	2 (12.5%)		16 (80.0%)	4 (20.0%)		10 (50.0%)	10 (50.0%)		4.9 (± 6.1)	
AutoG+S^7^	25 (35.7%)	19 (76.0%)	6 (24.0%)		20 (80.0%)	5 (20.0%)		18 (72.0%)	16 (88.9%)	2 (11.1%)		22 (88.0%)	3 (12.0%)		3 (12.0%)	22 (88.0%)		0.68 (± 2.2)	
AutoG+G^8^	3 (4.3%)	1 (33.3%)	2 (66.7%)		1 (33.3%)	2 (66.7%)		2 (66.7%)	2 (100.0%)	0 (0.0%)		3 (100.0%)	0 (0.0%)		0 (0.0%)	3 (100.0%)		0.0 (± 0.0)	

^1^Nonautologous graft, ^2^Autologous graft, ^3^Graft fixed with sutures, ^4^Graft fixed with glue only, ^5^Nonautologous graft fixed with sutures, ^6^Nonautologous graft fixed with glue only, ^7^Autologous graft fixed with sutures, ^8^Autologous graft fixed with glue only.

^a^Maximal perpendicular distance to graft measured on sagittal T2 MRI images (mm).

**Table 4:** Evolving syringomyelia on follow-up MRI depends on the kind of grafts and methods of graft fixation.

Graft and methods of graft fixation	Mean decrease in size of syringomyelia			
	Length -number of spinal segments (SD)	*p*	Cross-section area mm^2^ (SD)	*p*
Non-AutoG^a^	1.7 (± 2.99)	0.69	32.0 (± 44.31)	0.82
AutoG^b^	1.4 (± 3.18)		29.7 (± 35.11)	
S^c^	1.8 (± 3.06)	0.44	30.1 (± 35.10)	0.78
G^d^	1.2 (± 3.06)		33.0 (± 50.93)	

^1^Non-autologous graft, ^2^Autologous graft, ^3^Graft fixed with sutures, ^4^Graft fixed with glue.

Pseudomeningocele was present in 25 (35.7%) cases on follow-up MRI; however, it occurred significantly more frequently in the Non-AutoG group than in the AutoG group (52.4% vs. 10.7%; p < 0.05). Additionally, the rate of pseudomeningocele was similar in the G group compared with the S group (43.5% vs. 31.9%; p = 0.34). The rates of pseudomeningocele were 54.5%, 50.0%, and 12.0% in the Non-AutoG+S, Non-AutoG+G, and AutoG+S groups, respectively, and there were none in the AutoG+G group (p < 0.05). Correlations between duroplasty features and average thickness of pseudomeningocele are presented in Table 3.

Seven (10.0%) patients had postoperative complications: 4 (9.5%) in the Non-AutoG group and 3 (10.7%) in the AutoG group (p = 1, see Table S1 in supplementary material). Five (7.1%) patients needed revision surgery, and 2 were conservatively treated for aseptic meningitis (1 patient, Non-AutoG) and purulent cutaneous fistula (1 patient, AutoG). In the Non-AutoG group, 3 (7.1%) patients were reoperated on for cerebellar subsidence, of whom 2 (4.8%) also had large pseudomeningocele and 2 (4.8%) had acute hydrocephalus. In all patients with complications in the Non-AutoG group, the graft was fixed with glue (Non-AutoG+G). In the AutoG group, two patients (7.1%) required revision surgery: 1 (3.6%) for symptomatic extradural hematoma and 1 (3.6%) for symptomatic pseudomeningocele. All patients with complications in the AutoG group had the graft fixed with sutures (AutoG+S, Tables 3 and 5).

**Table 5:** Postoperative complications.

Complications	Number^a^ (%)	Duraplasty pattern (number)
Extradural hematoma	1 (1.4%)	AutoG+S (1)
Symptomatic pseudomeningocele	3 (4.3%)	Non-AutoG+G (2) AutoG+S (1)
Aseptic meningitis	1 (1.4%)	Non-AutoG+G (1)
Cerebellar subsidence	3 (4.3%)	Non-AutoG+G (3)
Acute hydrocephalus	2 (2.9%)	Non-AutoG+G (2)
Purulent cutaneous fistula	1 (4.3%)	AutoG+S (1)

^a^Seven (10%) patients had postoperative complications, with multiple adverse events in 3 of them: 1. cerebellar subsidence and large pseudomeningocele and acute hydrocephalus, 2. cerebellar subsidence and acute hydrocephalus, 3. cerebellar subsidence and large pseudomeningocele.

The risk of surgical revision for pseudomeningocele in the Non-AutoG, AutoG, S and G groups was 4.8%, 3.6%, 2.1% and 8.7%, respectively (Non-AutoG vs. AutoG: p = 0.81; S vs. G: p = 0.2). In the Non-AutoG+S, Non-AutoG+G, AutoG+S and AutoG+G groups, the risk of surgical revision was 0, 10.0%, 4.0% and 0, respectively (p = 0.34).

Three patients with pseudomeningocele underwent repeat duraplasty with suturing of the graft. All 3 cases of cerebellar subsidence were treated by resection of one cerebellar tonsil and by optimization of craniectomy size with the use of an artificial bone flap. One patient with acute hydrocephalus was treated with a ventriculoperitoneal shunt. Among patients with postoperative complications, the mean long-term CCOS score was 8.25 (SD ± 2.28) in the Non-AutoG group and 8.67 (SD ± 2.87) in the AutoG group.

## Discussion

For duraplasty, many autologous or nonautologous kinds of grafts have been described in the literature^7,10,17–20^. Furthermore, methods of graft fixation include suturing with running or single stiches or sealing with fibrin glue^7,21,22^. Klekamp reported an overall 73.6% clinical improvement in patients after posterior fossa decompression with duraplasty, in short-term follow-up regardless of whether autologous or nonautologous graft was used. Concurrently, he noted high neurological deterioration in long-term observation: 14.3% within 5 years and 15.4% within 10 years, with a significantly higher rate of symptom recurrence when the autologous graft was used^23^.

The average rate of syrinx cavity decrease after surgical decompression ranges between 76.1 and 81.1%^12,23–25^. Attenello et al*.* observed substantially better syrinx improvement in patients with nonautologous grafts (80.0%) than in patients with pericranial autografts (52.0%)^17^.

In our series, regardless of what kind of graft was used and how it was fixed, the clinical outcomes and effect on syrinx was very similar after a mean 5.3 years of follow-up. However, the AutoG+G subgroup was too small for reliable statistical comparisons (Table 3).

Pseudomeningocele formation was five times more frequent in our series when a nonautologous graft was used (52.4% vs. 10.7%, p < 0.05) and its mean maximal thickness was larger in the Non-AutoG group (4.62 mm vs. 0.61 mm, p < 0.05). Attenello et al*.* demonstrated a similar observation (pseudomeningocele occurrence: 24.0% vs. 11.0%)^17^.

Unexpectedly, of the method of graft fixation played a smaller role in our series than the type of graft. The risk of pseudomeningocele formation was 43.5% when the graft was fixed with glue and 31.9% in the when the graft was fixed with suturing (p > 0.05).

Most of the pseudomeningoceles were small, asymptomatic fluid spaces filling the dead extradural space, with no mass effect on the intradural structures and no subsequent healing problems or external CSF leaks. For greater accuracy in our study, we assessed every visible CSF collection above-graft on the MRI study, even if small (min. 2 mm of thickness), in order to verify their exact significance. However, they may cause psychological discomfort to the patients^26^. On the other hand, artificial grafts may present less potential to form strong adhesions with surrounding tissues on extra- and intradural side. This could explain why pseudomeningocele is more frequent and why some authors report better clinical outcomes with the use of artificial grafts^17,23^.

The most common complications reported in the literature were aseptic meningitis (4–32%), CSF leak (6–21%), and wound infection (0.5–7%)^5,23,24,27^. Some materials seem to predispose patients to specific complications, such as nonautologous grafts to aseptic meningitis and bacterial infections^10,27^. In our series, in the Non-AutoG group, one (2.4%) patient had aseptic meningitis, and in the AutoG group, one (3.6%) had a purulent cutaneous fistula. Vanaclocha observed CSF leakage in 15.0% of cases after duraplasty with a nonautologous graft (polytetrafluoroethylene) and none after pericranium usage^20^. Opposite results were reported by Klekamp and Attenello et al*.* observed with a higher rate of CSF leak after duraplasty with pericranium^17,23^. Observations similar to ours were reported by Yahanda et al. in a recent multi-center study where autografts and nonautologous grafts had a comparable complication rate (p = 0.12), with higher rates of pseudomeningocele (p = 0.04) related to the use of the nonautologous graft. However, methods of graft fixation were not evaluated and, unlike our adult cohort, concerned pediatric patients, which may have influenced the wound healing process^28^.

Complications occurred in 2 of our 4 duraplasty pattern groups, with no complications among the Non-AutoG+S and AutoG+G groups. However, differences in complication rates between the groups were statistically insignificant (Table 3). The highest complication rate was in the Non-AutoG+G group (20.0%); however, in 3 (15.0%) cases, the main indication for surgical revision was symptomatic cerebellar subsidence, which was due to an oversized craniectomy, rather than the duraplasty pattern. In general, neither the type of graft nor the method of its fixation were significantly associated with the risk and type of complications in our series.

To harvest the pericranium, an extension of the surgical approach is required, resulting in an increased risk of abnormal wound healing, such as infection or scar dehiscence, and cosmetic complications^10^. If the fascia lata is harvested, the scar on the thigh carries a potential risk of incisional muscle hernia and donor site hematoma; additionally, it could be cosmetically relevant because most patients are women. Abla et al*.* who compared autologous and non-autologous grafts, pointed out the lack of consensus regarding what kind of graft should be used; however, they recommend using pericranium if available in good condition^10^. The technique of pericranium harvesting without significant extension of the primary incision was described by Stevens et al*.*^29^ Vanaclocha et al*.* also advised the use of pericranium and described the disadvantages of other autologous grafts, such as ligamentous weakness of the neck after nuchal ligament harvesting^20^. In our opinion, each additional dissection in the proximity of the site of decompression creates a greater potential space for pseudomeningocele or hematoma formation. Non-autologous grafts have some advantages such as the ease of tailoring and reduction of operative time. Disadvantages are price, a higher risk of aseptic meningitis and pseudomeningocele formation.

Although pseudomeningocele occurred five times more frequently in our Non-AutoG group, it did not result in worse clinical results. On the other hand, we noted long-term occipitalgia worsening in every fourth patient after the use of their own pericranial flap. Harvesting of the pericranium may be associated with a greater risk of irritation or injury of the occipital nerves; however, the clinical relevance should be confirmed in further studies.

Our research presents limitations associated with its retrospective, single-center study design. Some patients had very long symptom durations and were in advanced stages of the disease before surgery. This may have influenced the clinical and radiological outcomes^30^. We are also aware that larger groups of patients have been published but most of these were children or children and adults together^28^. On the other hand, we had an opportunity to analyze and compare the results of 4 different duraplasty patterns applied on a homogenous cohort of patients for age, type of Chiari malformation, and the main principles of surgery technique for one center. To the best of our knowledge, the method of graft fixation in decompression surgery for CM-I has not been analyzed before. However, the relatively limited number of patients, particularly in the AutoG+G group with only 3 patients, did not allowed for firm conclusions. Besides, in the literature, a wide disparity of pseudomeningocele rate is published. Pseudomeningoceles incidentally recognized on imaging studies are considered by some researchers as asymptomatic findings, and do not, therefore, get reported as complications. Others, including our team, consider it important to also report all asymptomatic pseudomeningoceles^31^. A meta-analysis with multiple randomized prospective cohort studies would be necessary to draw a robust conclusion^32,33^. The potential causality needs to be explored further in the future by using the Mendelian Randomization framework or deep learning algorithms^34–40^.

## Conclusion

The occurrence of pseudomeningocele in CM-I decompression surgery was significantly higher when nonautologous grafts were used, regardless of the method of fixation. However, the vast majority of the pseudomeningoceles were asymptomatic, and there were no significant differences in long-term clinical outcomes, effect on syringomyelia or the risk of symptomatic postoperative complications. Thus far, our analysis indicates that autologous and nonautologous dural grafts can be either sutured or fixed with glue with very similar clinical results.

## Supplementary Information

Supplementary Information.
