# Supplementary material for: Comparison of dural grafts and methods of graft fixation in Chiari malformation type I decompression surgery
Source: Sci Rep. 2021 Jul 20;11:14801. doi: 10.1038/s41598-021-94179-4 (PMC8292506; doi:10.1038/s41598-021-94179-4)
Supplement: Supplementary file 1 — Supplementary Information. [file 41598_2021_94179_MOESM1_ESM.docx]

**Supplementary table**

| **Patient** | **Coexistence of post-operative complications** | **Duraplasty pattern** | **Treatment** | **Long-term outcome in CCOS^1^ (score)** |
| --- | --- | --- | --- | --- |
| 1. | Cerebellar subsidence,  pseudomeningocele, acute hydrocephalus | Non-AutoG+G^2^ | Revision surgery: implantation of artificial bone flap with resection of left tonsil and re-duraplasty (S) ^3^. | 8 |
| 2. | Cerebellar subsidence,  acute hydrocephalus | Non-AutoG+G | 1) Ventriculoperitoneal shunt implanted  2) Revision surgery: implantation of artificial bone flap with resection of right tonsil | 12 |
| 3. | Cerebellar subsidence,  pseudomeningocele, | Non-AutoG+G | Revision surgery: implantation of artificial bone flap with resection of left tonsil and re-duraplasty (S). | 7 |
| 4. | Symptomatic pseudomeningocele | AutoG+S^4^ | Re-duraplasty (S) | 9 |
| 5. | Extradural hematoma | AutoG+S | Evacuation of hematoma | 5 |
| 6. | Aseptic meningitis | Non-AutoG+G | Steroids | 6 |
| 7. | Purulent cutaneous fistula | AutoG+S | Antibiotics | 12 |

Tab. S1. Postoperative complications.

^1^ Chicago Chiari Outcome Scale,

^2^ Non-autologous graft fixed with glue,

^3^ Suturing,

^4^ Autologous graft fixed with sutures.
